# Data on links between structural and functional prokaryotic diversity in long-term sewage sludge amended soil

**DOI:** 10.1016/j.dib.2018.09.025

**Published:** 2018-09-14

**Authors:** Julen Urra, Itziar Alkorta, Iker Mijangos, Carlos Garbisu

**Affiliations:** a NEIKER-Tecnalia, Department of Conservation of Natural Resources, Soil Microbial Ecology Group, c/ Berreaga 1, E-48160 Derio, Spain; b Instituto BIOFISIKA (CSIC, UPV/EHU), Department of Biochemistry and Molecular Biology, University of the Basque Country, P.O. Box 644, 48080 Bilbao, Spain

## Abstract

The application of sewage sludge to agricultural soil induces co-exposure of prokaryotic populations to antibiotics and heavy metals, thus exerting a selection pressure that may lead to the development of antibiotic resistance. Here, soil samples from a long-term factorial field experiment in which sewage sludge was applied to agricultural soil, at different rates (40 and 80 t ha^−1^) and frequencies (every 1, 2 and 4 years) of application, were studied to assess: (i) the effect of sewage sludge application on prokaryotic community composition, (ii) the links between prokaryotic community composition and antibiotic resistance profiles, and (iii) the links between antibiotic resistance and metal(oid) concentrations in amended soil. We found no significant impact of sewage sludge on prokaryotic community composition. Some antibiotic resistance genes (ARGs) correlated positively with particular prokaryotic taxa, being *Gemmatimonadetes* the taxon with the greatest number of positive correlations at phylum level. No positive correlation was found between prokaryotic taxa and genes encoding resistance to sulfonamides and FCA. All metal(oid)s showed positive correlations with, at least, one ARG. Metal(oid) concentrations in soil also showed positive correlations with mobile genetic element genes, particularly with the gene tnpA-07. These data provide useful information on the links between soil prokaryotic composition and resistome profiles, and between antibiotic resistance and metal(oid) concentrations, in agricultural soils amended with sewage sludge.

**Specifications table**

**Table:** 

Subject area	*Biology*
More specific subject area	*Soil health, soil quality, soil resistome, prokaryotic functional diversity, prokaryotic structural diversity, heavy metals, metalloids, organic amendments, antibiotic resistance.*
Type of data	*Tables and Figures.*
How data was acquired	*Illumina MiSeq V2Platform; BioMark*^*™*^*HD System and Dynamic Array Integrated Fluidic Circuits (IFCs); Inductively Coupled Plasma Optical Emission-Spectrometry (ICP-OES, VARIAN).*
Data format	*Analyzed*
Experimental factors	*Sewage sludge was added to agricultural soil following a factorial design with combinations of two rates (40 & 80* *t* *ha*^−1^*) and three frequencies (every 1, 2 and 4 years) of application, as well as an unamended control.*
Experimental features	*16S rRNA metabarcoding was carried out following a dual indexing approach in an Illumina MiSeq V2 platform. Relative abundance of ARGs and MGE genes was measured by HT-qPCR. Heavy metal(oid) concentration in soil was determined by inductively coupled plasma-optical emission spectrometry (ICP-OES).*
Data source location	*Derio, Spain.*
Data accessibility	*Data are available in the article.*
Related research article	*J. Urra, I. Alkorta, I. Mijangos, L. Epelde, C. Garbisu, Application of sewage sludge to agricultural soil increases the abundance of antibiotic resistance genes without altering the composition of prokaryotic communities. Sci. Total Environ. 647 (2019) 1410–1420.*

**Value of the data**

• Data are useful for depicting the links between soil prokaryotic composition and antibiotic resistance profiles after long-term application of sewage sludge.
• Data are useful to show the long-term impact of sewage sludge application on the composition of prokaryotic communities in agricultural soil.
• Our data provide useful information on the links between metal(oid) concentrations in soil and the abundance of antibiotic resistance genes.

## 1 Data

**Fig. 1:**
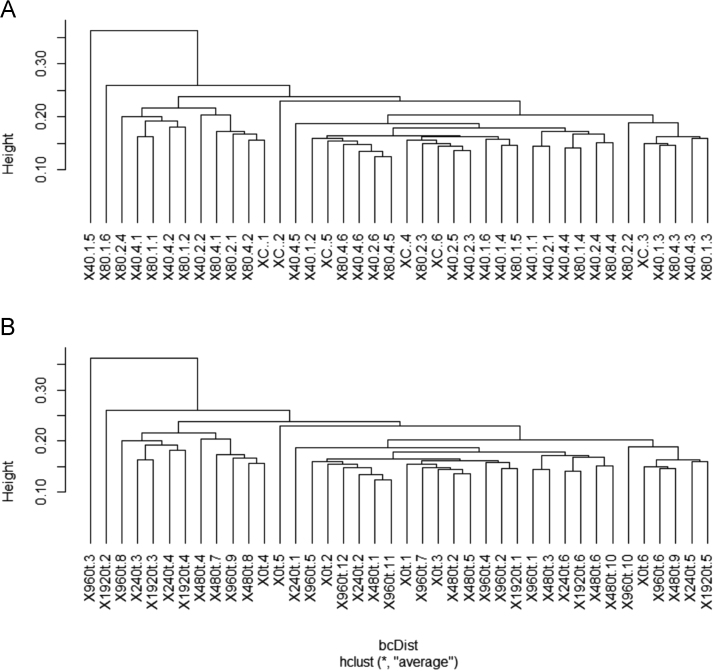
Hierarchical clustering of soil samples, based on Bray Curtis dissimilarities of prokaryotic OTUs obtained from 16S rRNA metabarcoding. Samples are arranged according to: (A) sewage sludge treatment; and (B) total amount of sewage sludge applied during the 24-year experiment. Treatments: 40-1: 40 t ha^−1^ every year; 40-2: 40 t ha^−1^ every 2 years; 40-4: 40 t ha^−1^ every 4 years; 80-1: 80 t ha^−1^ every year; 80-2: 80 t ha^−1^ every 2 years; 80-4: 40 t ha^−1^ every 4 years; C: control, unamended. Total amount of sewage sludge applied during the 24-year experiment (in t ha^−1^): 0, 240, 480, 960 and 1920.

**Fig. 2:**
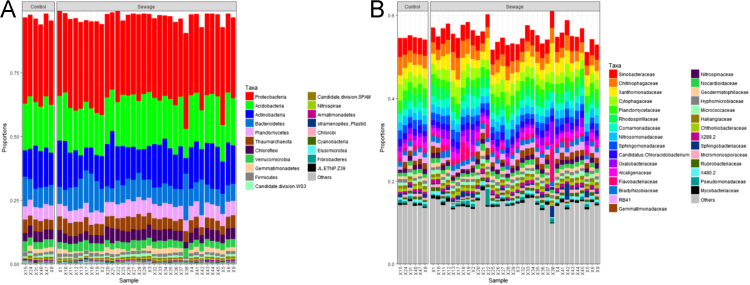
Barplots representing the composition of: (A) the 20 most abundant prokaryotic taxa at phylum rank; and (B) the 30 most abundant taxa at family rank, for all sewage-amended and unamended soil samples. Control: unamended samples. Sewage: sewage sludge amended samples.

**Fig. 3:**
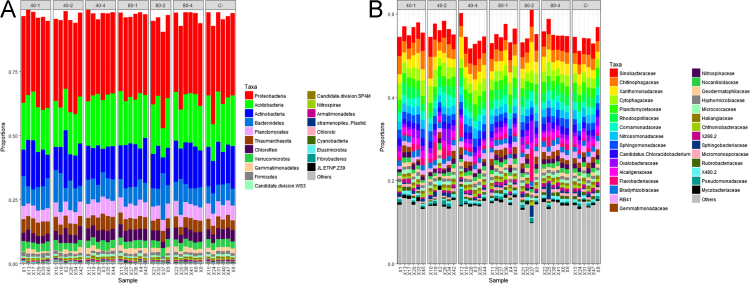
Effect of treatments on the composition of: (A) the 20 most abundant prokaryotic taxa at phylum rank; and (B) the 30 most abundant taxa at family rank. Treatments: 40-1: 40 t ha^−1^ every year; 40-2: 40 t ha^−1^ every 2 years; 40-4: 40 t ha^−1^ every 4 years; 80-1: 80 t ha^−1^ every year; 80-2: 80 t ha^−1^ every 2 years; 80-4: 40 t ha^−1^ every 4 years; C: control, unamended.

**Fig. 4:**
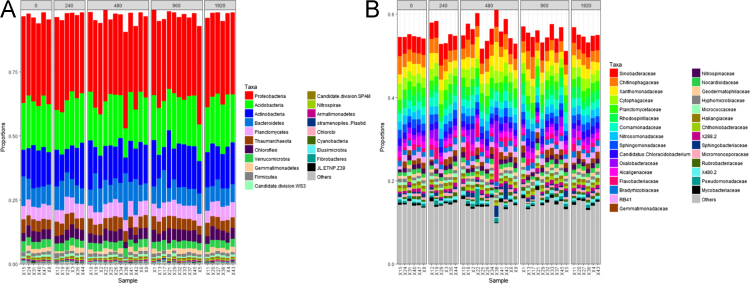
Effect of the total amount of sewage sludge applied during the 24-year experiment on the composition of: (A) the 20 most abundant prokaryotic taxa at phylum rank; and (B) the 30 most abundant taxa at family rank. Total amount of sewage sludge applied during the 24-year experiment (in t ha^−1^): 0, 240, 480, 960 and 1920.

We evaluated the effect of sewage sludge application on soil prokaryotic communities and the soil resistome, as reflected by the abundance of antibiotic resistance genes (ARGs) and mobile genetic element (MGE) genes. We investigated the correlations between ARG abundance and metal(oid) concentrations in soil. Hierarchical clustering did not show differences, neither among sewage sludge treatments (Fig. 1A) nor with respect to the total amount of sewage sludge applied to soil after 24 years (Fig. 1B). In relation to taxonomical classification, 96.6 and 55.3% of the 16S rRNA sequences were classified to phylum and family rank, respectively. At both levels, taxa distribution was similar among all soil samples and it did not show any significant differences (i) between sewage sludge amended *vs.* unamended soil (Fig. 2); (ii) among sewage sludge treatments (Fig. 3); and (iii) with respect to the total amount of sewage sludge added to the soil after 24 years (Fig. 4). The 10 most abundant prokaryotic taxa at phylum level accounted for 89.4% of the total community. Among them, *Proteobacteria*, the most dominant taxon, showed the greatest number of negative correlations (Table 1). No correlation was found between prokaryotic taxa and the abundance of ARGs for sulfonamides and FCA (Table 2). Several ARGs belonging to the other antibiotic groups studied here (aminoglycoside, β-lactamase, MLSB, tetracycline, vancomycin, multidrug) correlated with some prokaryotic taxa (Table 2). Concerning MGE genes, *Actinobacteria* and *Gemmatimonadetes* showed some positive correlations (Table 3). All metal(oid)s correlated positively with at least one ARG (Table 4). The gene *intI1* exhibited a significant negative correlation with Cu (Table 5). Most of the studied metal(oids) were positively correlated with, at least, one transposase encoding gene.

**Table 1:** Kendall׳s tau correlations between the 10 most abundant prokaryotic phyla. Negative correlation are displayed in italics.

	**Proteobacteria**	**Acidobacteria**	**Actinobacteria**	**Bacteroidetes**	**Planctomycetes**	**Chloroflexi**	**Verrucomicrobia**	**Gemmatimonadetes**	**Firmicutes**	**Candidate division WS3**
**Proteobacteria**		***	**	***	***	***	*	ns	ns	**
**Acidobacteria**	−*0.49*		ns	**	***	***	ns	ns	ns	***
**Actinobacteria**	−*0.32*	–		***	ns	***	***	***	ns	ns
**Bacteroidetes**	0.43	−*0.34*	−*0.69*		ns	***	***	***	ns	ns
**Planctomycetes**	−*0.45*	0.61	–	–		***	ns	ns	*	***
**Chloroflexi**	−*0.43*	0.37	0.37	−*0.41*	0.44		**	***	*	**
**Verrucomicrobia**	0.22	–	−*0.59*	0.50	–	−*0.32*		***	ns	ns
**Gemmatimonadetes**	–	–	0.49	*-0.45*	–	0.51	−*0.44*		**	ns
**Firmicutes**	–	–	–	–	−*0.24*	−*0.28*	–	−*0.29*		ns
**Candidate division WS3**	−*0.31*	0.55	–	–	0.63	0.29	–	–	–	

ns: not significant; *: *p* < 0.05; ** *p* < 0.01; *** p < 0.001.

**Table 2:** Kendall׳s tau significant correlations between the 10 most abundant prokaryotic phyla and the abundance of ARGs. Negative correlations are displayed in italics. Genes that were not amplified during the HT-qPCR analysis are highlighted in grey.

* *p* < 0.05; ** *p* < 0.01; *** *p* < 0.001. FCA: fluoroquinolone, quinolone, florfenicol, chloramphenicol and amphenicol resistance genes; MLSB: Macrolide-Lincosamide-Streptogramin B resistance.

**Table 3:** Kendall׳s tau significant correlations between the 10 most abundant prokaryotic phyla and the abundance of MGE genes. Negative correlations are displayed in italics.

		**Proteobacteria**	**Acidobacteria**	**Actinobacteria**	**Bacteroidetes**	**Planctomycetes**	**Chloroflexi**	**Verrucomicrobia**	**Gemmatimonadetes**	**Firmicutes**	**Candidate division WS3**
**Integrase**	**intI**							−0.34**	0.24*		
	**intI1**										
**Transposase**	**tnpA-03**										
	**IS613**										
	**tnpA-01**										
	**tnpA-04**										
	**tnpA-07**										
	**tnpA-05**										
	**Tp614**			0.26*							
	**tnpA-02**			0.25*					0.25*		

* *p* < 0.05; ** *p* < 0.01; *** *p* < 0.001.

**Table 4:** Kendall׳s tau significant correlations between metal(oid) concentration in soil and abundance of ARGs. Negative correlations are displayed in italics. Genes that were not amplified during the HT-qPCR analysis are highlighted in grey.

* *p* < 0.05; ** *p* < 0.01; *** *p* < 0.001. FCA: fluoroquinolone, quinolone, florfenicol, chloramphenicol and amphenicol resistance genes; MLSB: Macrolide-Lincosamide-Streptogramin B resistance.

**Table 5:** Kendall׳s tau significant correlations between metal(oid) concentrations in soil and the abundance of MGE genes. Negative correlations are displayed in italics.

		**Cu**	**Zn**	**Cd**	**Pb**	**Cr**	**Ni**	**As**
**Integrase**	**intI**							
	**intI1**	−0.34*						
**Transposase**	**tnpA-03**		0.39*					
	**IS613**							−0.36*
	**tnpA-01**							
	**tnpA-04**		0.36*					
	**tnpA-07**	0.45**	0.65***	0.39*			0.34*	
	**tnpA-05**				0.38*			
	**Tp614**							
	**tnpA-02**							

* *p* < 0.05; ** *p* < 0.01; *** *p* < 0.001.

## 2 Experimental design, materials, and methods

Experimental plots (35 m^2^; 6 replicates per treatment) were located in Navarre, Spain. A factorial design, with combinations of two rates (40 and 80 t ha^−1^) and three frequencies (every 1, 2 and 4 years) of sewage sludge (thermally dried and anaerobically digested) application, was followed in this field experiment. An unamended control was also included. Sewage sludge was annually (for 24 consecutive years) incorporated to the soil by disc plowing to a depth of 30 cm. The cropping system consists of 3-year crop rotations (cereal/cereal/non-cereal) with no irrigation or weed control [1]. Composite soil samples (i.e., 0–30 cm soil depth; 6 cores randomly taken per plot) were collected from each plot. The soil is a Calcaric Cambisol, with a clay-loamy texture, an alkaline pH (around 8.5) and an organic matter content of *ca.* 5%.

Soils were dried at room temperature and then sieved (<2 mm). Total concentrations of metal(oid)s (i.e. Cu, Zn, Cd, Pb, Cr, Ni, As) in soil samples were determined via inductively coupled plasma-optical emission spectrometry (ICP-OES). Samples for molecular analyses were stored at −20 °C. DNA was extracted from soil samples (3 soil aliquots per sample, 0.25 g DW soil each) using the Power Soil DNA Isolation Kit (MO Bio Laboratories, CA). Prior to DNA extraction, samples were washed twice in 120 mM K_2_PO_4_ (pH 8.0). Amplicon libraries were prepared with a dual indexing approach using sequence-specific primers targeting the V4 hypervariable region of the 16S rRNA gene according to the reaction mixtures, PCR conditions and primers described in Urra et al. [2]. Sequencing was carried out in an Illumina MiSeq V2 platform and pair-ended 2 × 250 nt. Merging of the read paired ends, quality filtering and clustering into operational taxonomic units (OTUs) was following Lanzén et al. [3]. Taxonomical assignments were carried out using CREST and SilvaMod v128 [4].

High-throughput RT-qPCR (HT-qPCR), with the nanofluidic qPCR BioMark^™^ HD system and 48.48 and 96.96 Dynamic Array Integrated Fluidic Circuits (IFCs) (Fluidigm Corporation), was used for the detection and quantification of ARGs and MGE genes in soil samples. 96 validated primer sets [5] targeting 85 ARGs, 10 MGEs and one reference structural gene (16S rRNA gene) were used. All samples were pre-amplified and treated with exonuclease I and then loaded onto the IFCs, following the Fluidigm׳s Fast Gene Expression Analysis Using EvaGreen Protocol. SsoFastTM EvaGreen® Supermix with Low ROX (Bio-Rad Laboratories, Redmond, WA) was used for amplification. The cycling program consisted of 1 min at 95 °C, followed by 30 cycles at 95 °C for 5 s and 60 °C for 20 s, followed by a melting curve. Data were then analyzed with the Fluidigm Real-Time PCR Analysis Software (v.3.1.3) with linear baseline correction and manual threshold settings, in order to obtain threshold cycle (Ct) values. Four technical replicates were included for each sample, and quantification of a specific gene was considered positive when 3 out of the 4 replicates were above the detection limit, which was established according to the lowest positive amplification value recorded in our experiment. Each ARG and MGE gene was normalized according to the matched structural reference gene (16S rRNA), in order to obtain a relative abundance value.

Metabarcoding data visualization and hierarchical clustering were performed with R package *vegan* [6]. Hierarchical clustering of prokaryotic OTUs was performed based on Bray-Curtis dissimilarity matrices to determine differences in soil prokaryotic composition between sewage sludge amended and unamended soil. In order to investigate the links between (i) the most dominant prokaryotic taxa, (ii) soil prokaryotic community composition and antibiotic resistance profiles, and (iii) total metal(oid) concentrations in soil and abundance of ARGs and MGE genes, Kendall׳s tau correlations were performed.
